# Inbreeding effects on different lineages of *Thrips tabaci* (Thysanoptera: Thripidae)

**DOI:** 10.1093/jisesa/ieae017

**Published:** 2024-03-14

**Authors:** Wondimagegn Atilaw Woldemelak

**Affiliations:** Department of Horticulture Science, College of Agriculture and Natural Resource Science, Debreberhan University, Debreberhan, Ethiopia

**Keywords:** brother and sister inbreeding, reproductive fitness, longevity, fecundity, egg hatchability

## Abstract

Inbreeding can have detrimental effects on reproductive fitness, but the extent of lineage-specific variation in these effects remains poorly understood. This study investigated the effects of brother and sister inbreeding on reproductive fitness in 2 lineages (L1 and T) of *T. tabaci*. Inbred females from both lineages exhibited a significant reduction in longevity compared with the control group. The L1 lineage experienced a 27% and 43% decrease in longevity in the F2 and F3 generations, respectively, while the T lineage showed a similar trend with a 30% and 44% decrease. The T lineage consistently displayed slightly longer lifespans than the L1 lineage across all generations. Brother and sister inbreeding also led to a decrease in fecundity rates in both lineages. In the F3 generation, the number of eggs laid decreased by 59% compared with the F2 generation. The T lineage consistently exhibited slightly lower fecundity rates compared with the L1 lineage. Egg hatchability rates declined with subsequent inbreeding, with the F3 generation showing lower rates compared with the F2 generation. However, the T lineage did not exhibit a significant difference in inbreeding depression for egg hatchability rates, while the L1 lineage demonstrated a noticeable decrease. Deformities observed in male L1 lineage resulting from inbreeding were consistent with disruptions in normal developmental processes, affecting various body parts such as legs, antennae, and wings. Continued inbreeding increased susceptibility to inbreeding depression in terms of longevity, fecundity, and egg hatchability.

## Introduction

Inbreeding is the mating between relatives carrying identical alleles (Dolgin et al. 2007) and is a common occurrence in small insect populations where access to unrelated mates is limited. However, in populations with higher density, inbreeding may not be expressed due to the availability of unrelated species to mate with. The consequences of inbreeding include a decrease in heterozygosity and a reduction in reproductive fitness in subsequent generations, collectively known as inbreeding depression (Hedrick and Garcia-Dorado 2016). Continued mating between close relatives leads to a decline in population size (Wright et al. 2008), making inbreeding depression particularly problematic in small populations that engage in successive inbred matings.

Frequent mating between relatives also contributes to an increase in the accumulation of deleterious mutations, a major factor driving extinction vortices that pose a threat to small populations. Inbreeding fosters homozygosity in the genome, allowing recessive deleterious alleles to impact various aspects of reproductive fitness, including fecundity, egg hatchability, immature survival, and adult longevity (White 2013). Diploid species are found to experience more intense inbreeding depression compared with haplodiploid species (Henter 2003). The variation in reproductive fitness can be attributed to the level of selection exerted on recessive deleterious alleles.

In haplodiploid species, the subsequent counterselection of deleterious alleles by haploid males significantly reduces the genetic load, leading to fewer recessive deleterious alleles compared with diploid species (Roff 2002, Charlesworth and Willis 2009). However, alleles of genes expressed exclusively in diploid females are protected from purifying selection in heterozygous individuals. In contrast, the genetic load hidden in heterozygous female diploids is expressed and purged in haploid males in haplodiploid species. This suggests that haplodiploids may suffer more pronounced inbreeding effects on life-history traits (Saito et al. 2000). Based on DNA sequences of the mitochondrial cytochrome *c* oxidase I (mt COI) genes, *Thrips tabaci* Lindeman, 1889, is currently classified into 3 lineages: 2 leek-associated lineages (L1, L2), and the tobacco-associated lineage (T) (Brunner et al. 2004, Toda and Murai 2007, Kobayashi and Hasegawa 2012, Jacobson et al. 2013, Fekrat et al. 2014). The leek-associated L1 represents the ancient form of *T. tabaci*. It is widely accepted that the tobacco-associated (T) lineage diverged from the ancient L1 lineage and subsequently adapted to solanaceous host plants (Brunner et al. 2004).

While the effects of inbreeding have been extensively studied in Coleoptera, Hymenoptera, Lepidoptera insect species, and various plant species, the impact of inbreeding on Thysanoptera insect species remains unexplored. As *T. tabaci* belongs to a haplodiploid insect pest species, it is plausible that mating between brother and sister may be the primary factor contributing to the observed effects on fecundity, egg hatchability rate, adult longevity, and the sex ratio of the progeny. Investigating the effects of inbreeding in Thysanoptera species, particularly in the lineages of *T. tabaci*, will provide valuable insights into the mechanisms underlying the impacts of inbreeding on reproductive fitness.

## Materials and Methods

### Specimen Collection

Specimens of *T. tabaci* from laboratory stock cultures were gathered, with the cultures having been established in 2013 and 2014. Thelytokous *T. tabaci* (L2) samples were acquired from various plants, such as *Filipendula vulgaris* Moench, *Santolina chamaecyparissus* L., *Lonicera caprifolium* L., *Disaphora Fruticosa* (L.) Rydberg, *Coriandrum sativum* L., *Stenactis annua* (L.) Persoon, and *Sorbaria sorbifolia* (L.) A. Braun, sourced from the Botanical Garden of Szent István University in Budapest. Additionally, samples were obtained from cabbage (*Brassica oleracea* L. convar. capitate var. alba) in central Hungary. Leek-associated arrhenotokous (L1) populations were gathered from onion bulbs in the traditional onion growing area of Makó, located in southern Hungary (46°14ʹ N, 20°28ʹ E, 76 m altitude). Tobacco-associated arrhenotokous (T) populations were procured from tobacco fields in Apagy (47°57ʹ N, 21°55ʹ E, 118 m altitude), Pócspetri (47°52ʹ N, 21°59ʹ E, 133 m altitude), and Encsencs (47°44ʹ N, 22°06ʹ E, 153 m altitude) in East-Hungary.

### Experimental Design

#### Sample collection and rearing

To initiate the experiment, 24 female adults were selected from the stock colonies of L1 and T lineages. Each female was isolated and reared individually in separate 2-ml microcentrifuge tubes. The tubes were equipped with cabbage leaf discs as a food source for L1 lineage and tobacco leaf discs for T lineage. The isolated individuals were maintained under controlled environmental conditions, maintaining a temperature of 23 °C and a long daylight period of 16 h of light followed by 8 h of darkness.

#### Lineage confirmation

After the death of each isolated female, individual preservation in 96% ethanol served as a means for subsequent lineage confirmation. Lineage confirmation was conducted through a thorough examination of the mitochondrial COI (mtCOI) product sequences. Established methods and protocols, as described by (Farkas et al. 2019), were followed to ensure accurate identification and confirmation of the specific lineage, whether it belonged to the L1 or T T. tabaci lineages. The lineage confirmation step added an important layer of accuracy to our experimental design by ensuring that the correct individuals from the desired lineages were used in subsequent analyses.

#### Sample collection and preparation

Individual female and male adult thrips specimens of known origin and reproductive mode were selected for DNA isolation. These thrips were transferred from a 2-ml microcentrifuge tube to separate 1.5-ml microcentrifuge tubes using a micropipette and a fine brush. Prior to further processing, any residual ethanol present on the thrips’ integument was allowed to evaporate.

#### DNA extraction

Genomic DNA extraction from the thrips specimens was performed using a modified version of the rapid standard method incorporating Proteinase K treatment, as described by Farkas et al. (2019). In a dedicated 1.5-ml microcentrifuge tube, the thrips were homogenized in 7 µl of tissue lysis buffer. The tissue lysis buffer contained 10 mM Tris–HCL (pH 8.4), 50 mM KCL, 0.45% Tween 20, 0.2% gelatin, and 0.45% Nonidet P-40. To facilitate efficient protein digestion, 3 µl of Proteinase K enzyme (at a concentration of 60 µg/ml) sourced from ThermoFisher Scientific, Waltham, Massachusetts, USA, was added to the crushed thrips sample. The resulting lysate was thoroughly mixed using a vortex and subsequently centrifuged at 13,000 rpm for 30 s. The obtained homogenate was subjected to an incubation period of 30 min at 65 °C, ensuring complete protein digestion within the sample. Following this, the sample was incubated at 95 °C for 15 min to effectively inactivate the Proteinase K enzyme, rendering it nonfunctional. The isolated genomic DNA was meticulously resuspended in 20 µl of TE buffer, which consisted of 10 mM Tris–HCL and 1 mM EDTA at a pH of 8.0. The DNA solution was mixed to guarantee proper resuspension, and no further processing steps were undertaken. The final DNA extracts were properly stored at −20 °C, maintaining their stability and integrity until subjected to subsequent analyses. To differentiate between species, the mtCOI gene was selected in this study. The PCR primers utilized were custom designed based on the available *T. tabaci* target sequences found in the NCBI GeneBank nucleotide database (https://www.ncbi.nlm.nih.gov/genbank/). The specific forward primer was named “TTL-UNIF1” (5ʹ-ATTAATTATAGGRCTTTAYAAAGAAGG-3ʹ), and the reverse primer was called “TTL-UNIR1” (5ʹ-GTAGTGAAAGTGAGCTACAACATAATA-3ʹ). To amplify the target gene, a 20-µl PCR mix was prepared, which comprised 10 µl of 2 × DreamTaq Green PCR Master Mix (ThermoFisher Scientific, Waltham, Massachusetts, USA), 20–80 ng of DNA template (1 µl), 0.5 mM (1 µl) of each primer, and nuclease-free water to make a total volume of 20 µl. The PCR protocol involved an initial denaturation step at 95 °C for 1 min, followed by 40 cycles of denaturation at 95 °C for 1 min, annealing at 53 °C for 1 min, extension at 72 °C for 1 min, and a final extension at 72 °C for 10 min. The resulting amplified product had a size of 780 bp. The PCR was conducted using an Eppendorf Mastercycler Nexus Gradient Thermal Cycler (Eppendorf AG, Hamburg, Germany). For gel electrophoresis, a 2.5% agarose gel was utilized, along with a 100 bp DNA ladder (ThermoFisher Scientific) as a standard marker. The amplified DNA fragment was visualized under a UV transilluminator and documented using a gel documentation system (UVP BioDoc-It Imaging Systems LMS-20E, Upland, California, USA).

#### Progeny production and parental generation (F1)

Following the preservation of each deceased female in 96% ethanol, the newly hatched progeny resulting from each individual were collected and reared individually until they reached adulthood. These raised individuals constituted the parental generation, referred to as F1, which served as a crucial control group for subsequent comparisons. During the rearing process, individuals from both the L1 and T lineages were treated under the same conditions and received identical care and nutrition.

#### Inbreeding coefficient determination and subsequent generations (F2 and F3)

To specifically investigate the effects of inbreeding depression, subsequent generations beyond the F1 parental generation were established. In the case of haplodiploid Thysanoptera species like *T. tabaci*, the inbreeding coefficient for female offspring resulting from full-sibling mating is equivalent to that of diploids, as established by Wright (1969).

To replicate this scenario, controlled mating in F_2_ and F_3_ generations involved full-sibling crosses, where individuals from the same lineage and generation were selected as mating partners. This deliberate mating strategy allowed for the evaluation of the effects of inbreeding on lifetable parameters and reproductive fitness. The inbreeding coefficient for the resulting inbred haplodiploid females was calculated based on the well-established understanding that in haplodiploids, the inbreeding coefficient is the same as that of diploids. For the F_2_ generation, the inbreeding coefficient was calculated to be 0.25, indicating a moderate level of inbreeding. In the subsequent F_3_ generation, the inbreeding coefficient increased to 0.375, reflecting a progressively higher level of inbreeding. These determined inbreeding coefficients serve as indicators of the degree of relatedness between mating individuals and provide insights into the level of inbreeding within the subsequent generations.

#### Assessment of inbreeding depression

In order to measure the effects of inbreeding depression, controlled crosses were conducted within the L1 and T lineages. These crosses involved specific pairings between brother and sister pairs, representing varying degrees of kinship. Fitness parameters may include survival rates, reproductive output, and overall fitness, while lifetable parameters encompass various aspects such as generation time, age-specific survival rates, and fertility rates.

Through the analysis of these parameters, the effects of brother and sister inbreeding depression on the studied lifetable parameters and reproductive fitness could be assessed and quantified. By comparing the performance of the offspring resulting from different kinship pairings, such as brother and sister inbreeding, can gain insights into the intensity and nature of inbreeding depression within the L1 and T lineages.

#### Egg production

To ensure the production of sufficient eggs for the treatments, female specimens were randomly chosen from the progeny of the preceding generation and placed in separate 2-ml microcentrifuge tubes. These specimens were provided with cabbage and tobacco leaf discs as both a food source and oviposition substrate. They were kept at a consistent temperature of 23 °C under prolonged daylight conditions (16 h of light, 8 h of darkness). The leaf discs were changed every 48 h, and the eggs within the leaf tissue were counted using the bottom light of a stereomicroscope (Alpha, NSZ-606, Novel Optics, Ningo Yongxin, China). Upon hatching, the first-instar nymphs were isolated individually and raised in isolation until adulthood. In order to ensure mating between siblings of the L1 and T lineages, a newly emerged F1 brother and sister, both produced from the same mother, were confined in the same microcentrifuge tube for 48 h. After this period, the male was removed, and the female was kept isolated individually for the rest of its lifetime.

#### Progeny rearing

The newly hatched progeny from the preserved females were raised individually to adulthood. These progenies served as the parental generation (F1) for the subsequent inbred line. In the F1 generation, the inbreeding coefficient was assumed to be zero, serving as a control for comparison with the F2 and F3 generations. For F2 and F3 generations, it was assumed that the inbreeding coefficient was greater than one.

By following this experimental design, this research aim to unravel lineage-specific variation in susceptibility to inbreeding effects on reproductive fitness in onion thrips (*Thrips tabaci*) by studying lifetable parameters and fitness measures in different generations and inbred lineages.

##### Treatments

To study the effects of brother and sister inbreeding, a specific experimental design was followed for 3 consecutive generations. In this design, females were randomly chosen from the progeny of the previous generation.

##### First generation (F1)

The experiment commenced with the F1 generation. To form this generation, females were randomly selected from the progeny of the previous generation. This step ensured that the females in the F1 generation were closely related to one another.

##### Second generation (F2)

The F2 generation comprised females that were the daughters of the F1 females. To produce the females of the F2 generation, a deliberate brother and sister mating strategy was implemented. The F1 females, randomly selected from the previous generation, were mated with their brothers from the same mother.

#### Population Lifetable Parameters

The following key lifetable parameters, such as the intrinsic rate of increase (*r*) and the finite rate of increase (λ), were computed using the procedures outlined by Nielsen et al. (2010).

### Malformations of the Progeny

To investigate the malformations observed in the progenies of L1 and T lineages resulting from inbreeding and analyze disruptions in normal developmental processes, a controlled breeding experiment was designed utilizing a selected population of L1 and T lineages. In order to achieve a high degree of genetic relatedness, deliberately chosen inbred mating pairs were utilized. The subsequent offspring from these mating pairs were monitored throughout their developmental stages to identify any indications of abnormalities or phenotypic variations. An examination of the affected body parts, such as legs, antennae, and wings, was conducted through thorough visual inspections using the bottom light of a stereomicroscope (Alpha, NSZ-606, Novel Optics, Ningo Yongxin, China). Diligent documentation of the observed deformities was performed, and these findings were subsequently compared with control groups. The primary objective of this analysis was to evaluate the extent of disruptions in normal development resulting from inbreeding.

### Statistical Analysis

For the statistical analysis of the data, IBM SPSS 25 (SPSS Inc., Chicago, Illinois, USA) was used. To test the hypothesis that there would be mean differences in inbreeding depression effects on different generations, a general linear model of univariate analysis of variance was employed. This analysis was conducted separately for female longevity, fecundity, and egg hatchability rate. All reported means are accompanied by their respective standard deviations. Prior to conducting the analyses, the data were checked for normality using nonparametric Kolmogorov–Smirnov and Shapiro–Wilk tests (*P* > 0.05). Skewness and kurtosis were also examined. It should be noted that the normality assumption was violated for the data related to egg hatchability. To rectify this issue and normalize the distribution, the variable was transformed using an arcsine transformation. Before conducting a series of follow-up *t*-tests, the homogeneity of variance assumption was assessed using Levene’s test, which was found to be acceptable. Finally, the independent samples *t*-test was applied to compare significant differences in the variables. This comprehensive statistical analysis allowed for a rigorous examination of the data and ensured the accuracy of the reported findings.

## Results

### Lineage Confirmation

Based on the analysis of the mtCOI region, it was observed that the genetic makeup of 20 mothers belonged to L1 and T lineages. As expected, these mothers were found to genetically align with their respective lineages. Specifically, the L1 lineage demonstrated 3 distinctive DNA fragments after the digestion process. The sizes of these fragments were measured to be 345, 274, and 161 bp. On the other hand, the T lineage displayed only one DNA fragment on the DNA ladder, with a size of 780 bp.

### Effects of Brother and Sister Inbreeding on Longevity

**Fig. 1.**
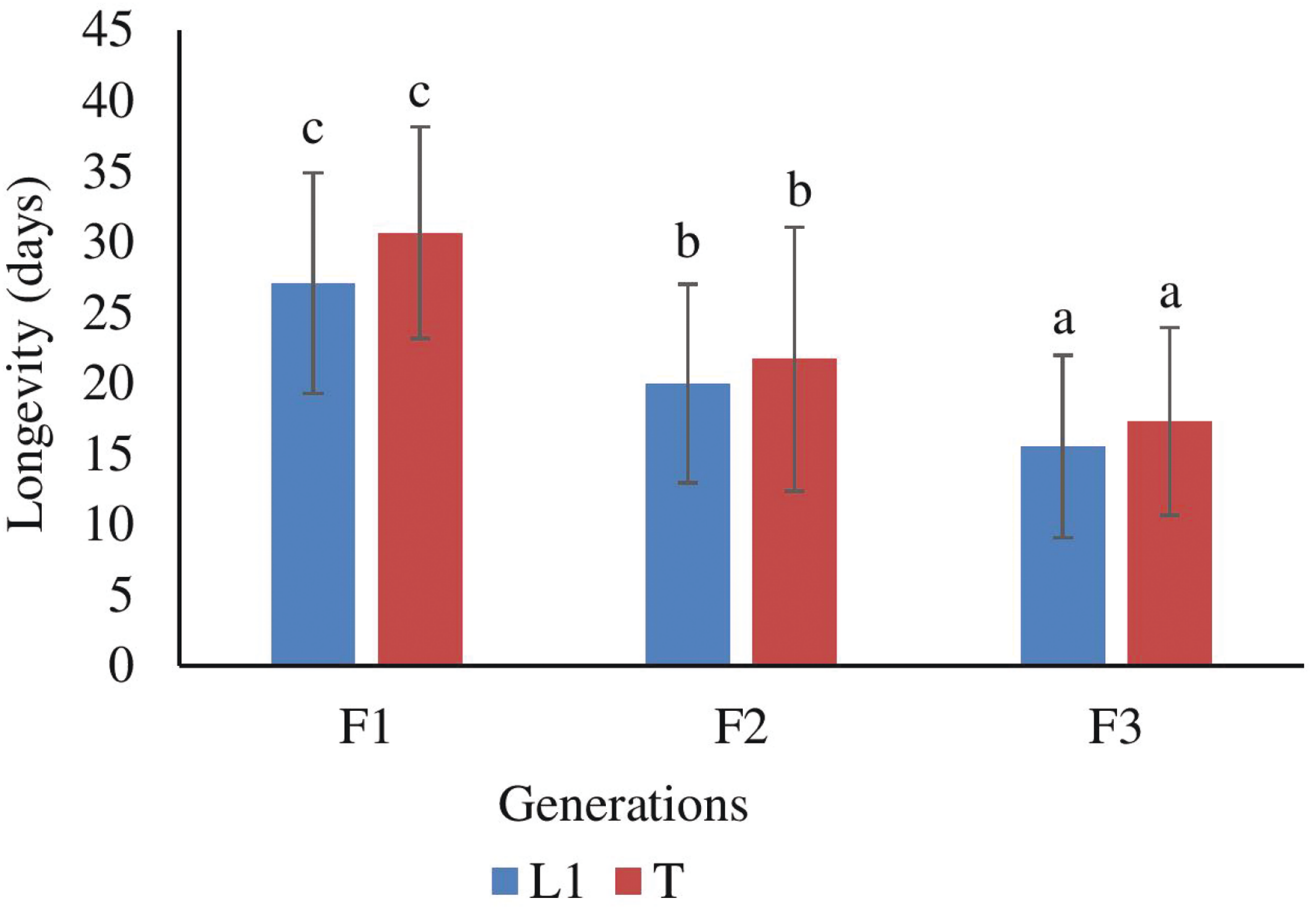
The comparison of brother and sister inbreeding on longevity at different generations. Different letters indicate significant difference with *P* < 0.05. Error bars indicate SE.

The effects of brother and sister inbreeding on longevity were assessed in the L1 and T lineages, and significant differences were observed. Statistical analysis, including *F*-tests, revealed significant differences in longevity between different generations of brother and sister inbreeding for both lineages (*F*(2, 139) = 31.491, *P* < 0.001 for L1 lineage; *F*(2, 134) = 38.79, *P* < 0.001 for T lineage). The results showed that inbred females from both lineages had significantly shorter lifespans in the F2 and F3 generations compared with females in the F1 generation. Additionally, the longevity of both lineages was significantly lower in the F3 generation compared with the F2 generation. Specifically, there was a 27% and 43% reduction in longevity for the L1 lineage in the F2 and F3 generations, respectively. Similarly, the T lineage experienced a 30% and 44% reduction in longevity in the F2 and F3 generations, respectively, when compared with the females of the F1 generation. These findings strongly indicate that as brother and sister inbreeding continues, there is an increased susceptibility to inbreeding depression, specifically in terms of longevity. Notably, the T lineage exhibited slightly longer lifespans in all generations compared with the L1 lineage (Fig. 1). This detailed analysis provides robust evidence for the negative impact of continued brother and sister inbreeding on longevity, emphasizing the importance of genetic diversity in maintaining healthy populations.

### Effects of Brother and Sister Inbreeding on Fecundity

**Fig. 2.**
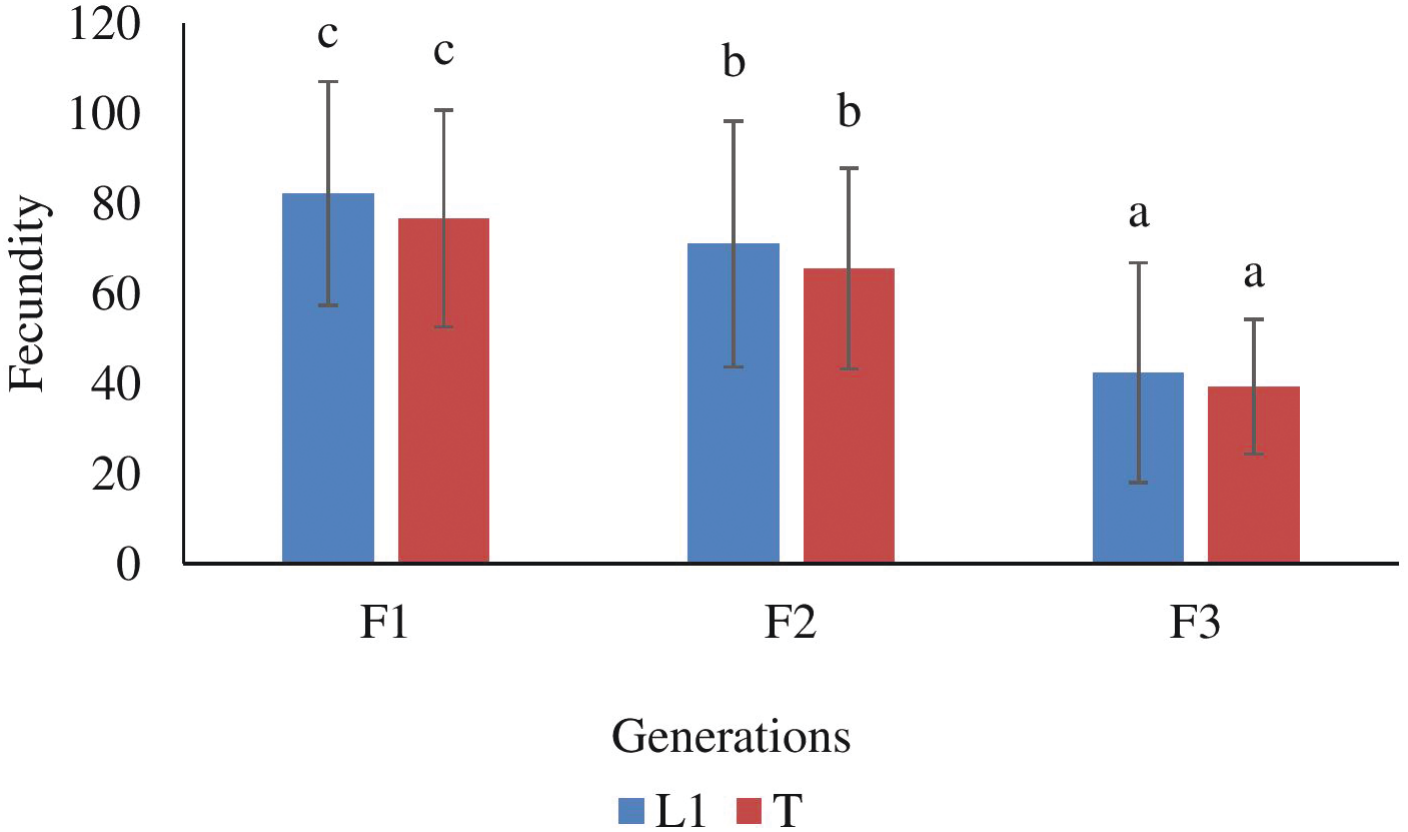
The comparison of brother and sister inbreeding on fecundity at different generations. Different letters indicate significant difference with *P* < 0.05. Error bars indicate SE.

The fecundity rates of the L1 and T lineages were examined in relation to different generations of brother and sister inbreeding, yielding significant differences (*F*(2,134) = 39.142, *P* < 0.001 for L1 lineage; *F*(2, 139) = 29.549, *P* < 0.001 for T lineage). In both lineages, a noticeable reduction in fecundity was observed starting from the F2 generation, and this reduction became even more prominent in the F3 generation. Specifically, in the F3 generation, the inbred females of both lineages laid 59% fewer eggs compared with the inbred females in the F2 generation. It is worth mentioning that the fecundity of the T lineages was slightly lower than that of the L1 lineage in all generations (Fig. 2). This suggests that subsequent brother and sister inbreeding led to a higher magnitude of depression in fecundity for both the L1 and T lineages. These findings provide compelling evidence that continued brother and sister inbreeding has a substantial impact on fecundity, resulting in a significant reduction in the number of eggs laid by the inbred females. It emphasizes the negative consequences of inbreeding and indicates the importance of maintaining genetic diversity for maintaining optimal reproductive output in populations.

### Effect of Brother and Sister Inbreeding on Egg Hatchability

**Fig. 3.**
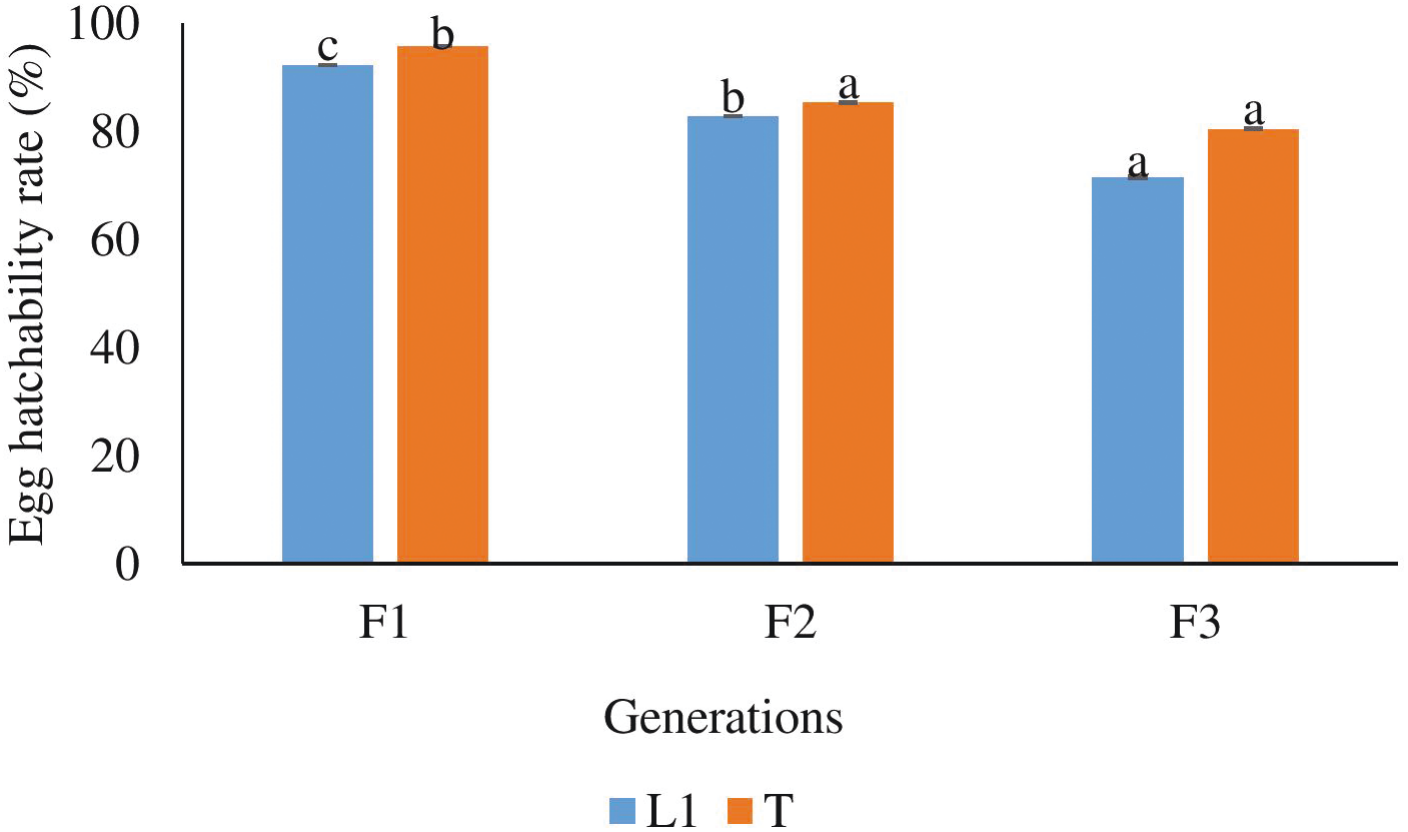
The comparison of brother and sister inbreeding on egg hatchability rate at different generations. Different letters indicate significant difference with *P* < 0.05. Error bars indicate SE.

The egg hatchability rates of both the L1 and T lineages were examined in relation to different generations of brother and sister inbreed significant differences were found (*F*(2, 134) = 69.299, *P* < 0.001 for L1 lineage; *F*(2, 139) = 36.128, *P* < 0.001 for T lineage). For both lineages, a decline in egg hatchability rate was observed starting from the F2 generation. There were significant differences in egg hatchability rates between the F2 and F3 generations for the L1 lineage, indicating a decrease in hatchability with subsequent inbreeding. However, no significant difference in inbreeding depression was observed for the T lineage. In the F2 generation, more than 82% of the eggs hatched in both lineages, indicating a high hatchability rate. However, in the F3 generation, the hatchability rates decreased to less than 72% for the L1 lineage and less than 80% for the T lineage. It is noteworthy that the T lineages exhibited significantly better egg hatchability rates in the F1 and F2 generations compared with the L1 lineage (Fig. 3). These findings further highlight the negative consequences of continued brother and sister inbreeding, as evidenced by the decrease in egg hatchability rates observed in subsequent generations. The results emphasize the importance of genetic diversity in maintaining optimal reproductive success in populations.

#### Malformations of the progeny

The results of the study unveiled compelling evidence regarding the impact of brother and sister inbreeding depression on normal developmental processes. The findings demonstrated that this practice resulted in significant disruptions, ultimately leading to a range of malformations affecting various body parts in the offspring. Specifically, the antennae exhibited notable asymmetry, with deviations from the expected balanced structure. This asymmetry in antennae length and shape indicated aberrations in their growth and development. The legs of the affected individuals displayed variations in length, deviating from the typical uniformity observed in noninbred populations. These variations are sometimes manifested as legs appearing longer or shorter than the anticipated norm. Additionally, the study highlighted irregularities in the development and functioning of wings. The wings of inbred individuals exhibited irregular patterns of spreading, implying compromised flight capabilities. These irregular patterns indicated improper development and growth of wing tissues, likely linked to disruptions in the intricate processes associated with wing formation. The observed malformations in the wings further solidified the impact of brother and sister inbreeding on normal developmental processes.

### Population Growth Parameters

The findings from this laboratory work indicate that both lineages, L1 and T, are experiencing a decline in population based on the intrinsic rate of increase (*r*_m_) and the finite rate of increase (λ). Specifically, the intrinsic rate of increase (*r*_m_) for the L1 lineage was −0.432, while for the T lineage, it was −0.360. This suggests that the populations of both lineages are decreasing. Additionally, the finite rate of increase (λ) for the L1 and T lineages was measured to be 0.648 and 0.698, respectively. This indicates that the populations of L1 and T lineages are expected to decline by 35.2% and 30.2% per generation, respectively.

## Discussion

The present study investigated the effects of brother and sister inbreeding on reproductive parameters, including longevity, fecundity, and egg hatchability, in the L1 and T *T. tabaci* lineages. The results consistently revealed the presence of inbreeding depression across all the traits examined, highlighting the detrimental consequences of continued inbreeding on reproductive success in these lineages.

In terms of longevity, significant reductions were observed in the lifespan of inbred females compared with those from the F1 generation, indicating the harmful effects of inbreeding on individual survival. This reduction in lifespan can be attributed to the accumulation of harmful recessive alleles resulting from close familial mating (Henter 2003). Furthermore, the decrease in lifespan was found to be progressive, with a substantial decrease observed in the F3 generation, highlighting the cumulative negative impact of continued inbreeding. High inbreeding effect on the longevity (38% longevity reduction) has been reported in *Uscana semifumipennis* (Girault, 1911) (Hymenoptera: Trichogrammatidae) (Henter 2003). It is worth noting that slightly longer lifespans were observed in the T lineage compared with the L1 lineage across all generations, suggesting some variation in susceptibility to inbreeding effects between lineages. The observation of slightly longer lifespans in the T lineage compared with the L1 lineage across all generations suggests that there may be variation in susceptibility to inbreeding effects between these lineages. The T lineage seems to exhibit a higher resistance to these negative effects, resulting in longer lifespans compared with the L1 lineage. It is worth noting that this observation indicates a correlation between lineage and lifespan, but it does not necessarily imply causation. Other factors, such as genetic differences or environmental influences, might also contribute to the observed variation in lifespan. Further research is needed to fully understand the underlying reasons for these differences in susceptibility to inbreeding effects between lineages.

The study also revealed a reduction in the fecundity of inbred females as brother–sister inbreeding continued. The magnitude of this reduction was highlighted by the 59% decrease in the number of eggs laid in the F3 generation compared with the F2 generation. This decline in fecundity further supported the idea that inbreeding leads to the expression of deleterious genetic traits and reduced reproductive fitness (Saito et al. 2000). Lifetime productivity shows high levels of inbreeding depression in *Drosophila simulans* (Sturtevant, 1913) (Diptera: Drosophilidae) (Wright et al. 2008). The reduction in fecundity observed in female *Callosobruchus maculatus* (Fabricius, 1775) (Coleoptera: Chrysomelidae) due to inbred males can be attributed to a decreased number of sperm in their ejaculates (Fox et al. 2011, Mehlis et al. 2012). The reduced quality of sperm in inbred males can manifest as abnormalities in sperm morphology, reduced motility, or compromised genetic integrity (Mehlis et al. 2012). When inbred males mate with females, the lower quality sperm may have difficulty fertilizing eggs effectively, leading to impaired embryonic development. This can result in decreased hatching success and lower overall reproductive success for the resulting offspring. Furthermore, inbreeding can also lead to reduced female fertility (Michalczyk et al. 2010). The negative effects on sperm quality in inbred males may have a direct impact on the number of eggs that females are able to produce after storing the sperm for extended periods. This reduced female fertility further contributes to the negative consequences of inbreeding on reproductive fitness. Interestingly, slightly lower fecundity was observed in the T lineage compared with the L1 lineage across all generations, indicating a higher magnitude of depression in fecundity for the T lineage. The observed lower fecundity in the T lineage compared with the L1 lineage across all generations could be attributed to the effects of brother and sister inbreeding. Inbreeding, which involves mating between closely related individuals, can lead to a reduction in genetic diversity and an increased risk of harmful genetic mutations. Over successive generations of inbreeding, these negative effects can accumulate, resulting in decreased reproductive success and lower fecundity. The T lineage might have experienced a higher magnitude of brother and sister inbreeding compared with the L1 lineage. This sustained inbreeding may have led to the accumulation of detrimental genetic variations, impacting the fecundity of individuals within the T lineage. It is important to note that this explanation is based on the assumption that inbreeding is the cause of the observed difference in fecundity. Further research would be necessary to confirm this hypothesis and to explore other potential factors that could contribute to the lower fecundity in the T lineage.

Regarding egg hatchability, the study found a progressive decline in hatchability rates with subsequent generations of brother–sister inbreeding. This decline can be attributed to the accumulation of harmful recessive alleles and reduced genetic diversity resulting from inbreeding (Akimoto 2007). Additionally, significant differences in hatchability rates were observed between the F2 and F3 generations in the L1 lineage, providing further evidence of inbreeding depression. Severe inbreeding depression in egg hatchability rates has been reported in *Lymantria dispar* (Linnaeus, 1758) (Lepidoptera: Erebidae) (Higashiura et al. 1997). In contrast to the L1 lineage, no significant inbreeding depression in egg hatchability rates was observed in the T lineage. This suggests that there may be genetic or ecological factors influencing the susceptibility to inbreeding effects among different lineages, which require further investigation to better understand the underlying mechanisms. The absence of significant inbreeding depression in egg hatchability rates in the T lineage, as opposed to the L1 lineage, suggests that there may be genetic or ecological factors influencing the susceptibility to inbreeding effects among different lineages. Genetic factors could play a role in determining the ability of individuals within a lineage to cope with the negative effects of inbreeding. It is possible that the T lineage has developed genetic variations or adaptations that enable it to withstand inbreeding without a significant decrease in egg hatchability rates. These genetic factors could contribute to the maintenance of reproductive fitness even in the face of close mating (Doreswamy and Gopal 2012). Environmental conditions, resource availability, or specific interactions within the breeding population could influence the impacts of inbreeding on egg hatchability. The T lineage may be exposed to different ecological conditions that mitigate the negative effects of inbreeding, resulting in comparable hatchability rates to outbred individuals. To better understand the underlying mechanisms and the specific factors at play, further investigation is needed. Detailed genetic analyses, studies on environmental influences, and controlled experimental designs could provide insights into why different lineages exhibit varying susceptibility to inbreeding effects on egg hatchability rates.

Deformities observed in male onion thrips as a result of brother and sister inbreeding can be attributed to various genetic and developmental factors. Inbreeding can lead to a higher expression of deleterious recessive genes and a reduction in genetic diversity within a population. As a consequence, harmful genetic mutations that would usually be masked in outbred populations can become more apparent in inbred individuals. The deformities observed in the legs, antennae, wings, and other body parts of male onion thrips can be explained by disruptions in normal developmental processes. This phenomenon has been observed in various insect species (Akimoto 2014). Inbreeding increases the chances of inheriting 2 copies of a deleterious gene, which can interfere with the proper formation and development of these body parts. For example, mutations in genes responsible for limb development can result in leg deformities, while abnormalities in genes involved in wing development can lead to wing deformities.

The mechanisms through which the occurrence of genetic deformities happens are complex. Inbreeding can disrupt genetic processes, such as recombination, and increase the expression of homozygous recessive alleles, which may have detrimental effects on development (Møller et al. 2005). As a consequence, the accumulation of harmful mutations across generations can lead to an increased frequency of deformities in inbred individuals. Further research is necessary to gain a more comprehensive understanding of the specific genes and mechanisms underlying the deformities observed in male onion thrips due to brother and sister inbreeding. Investigating the genetic basis of these deformities could help identify potential markers for assessing the genetic health and viability of onion thrips populations, contributing to the development of effective strategies for their management and conservation.

In conclusion, this study provides compelling evidence for the presence of inbreeding depression on various reproductive parameters in the L1 and T lineages. The negative impacts of continued brother–sister inbreeding on longevity, fecundity, and egg hatchability underscore the importance of genetic diversity for maintaining healthy and robust populations.
